# Epidemiological studies on Dermatophytosis in human patients in Himachal Pradesh, India

**DOI:** 10.1186/2193-1801-3-134

**Published:** 2014-03-09

**Authors:** Vikesh Kumar Bhatia, Prakash Chand Sharma

**Affiliations:** Department of Microbiology, Shoolini University of Biotechnology and Management Sciences, Bajhol, Solan, Himachal Pradesh India; Superannuated as Professor Microbiology, Veterinary Microbiology, Haryana Agricultural University, Hisar, Haryana India

**Keywords:** Dermatophytosis, Superficial mycoses, Tinea, *Trichophyton spp*, *Microsporum spp*

## Abstract

Dermatophytes are among the common fungal agents implicated in superficial skin infections worldwide. They include species of *Trichophyton, Microsporum* and *Epidermophyton.* In hot and humid climates of tropical and subtropical regions, the incidence of these pathogens is higher. We present in this article, the epidemiological data regarding the prevalence of different dermatophyte species involved in superficial mycoses in human patients in the state of Himachal Pradesh (India) and different clinical conditions, age and sex of the patients. A total of 202 samples in the form of skin and nail scrapings, hair follicles were collected from different ringworm/tinea conditions which included: Tinea corporis, T. capitis, T. cruris, T. pedis, T. unguium, T. faciei, T. manuum and T. gladiatorum. On culturing, 74 samples (36.6%) were found positive for dermatophyte spp. *Trichophyton spp*. was the predominant one (98.65% cases) followed by *Microsporum gypseum* (1.35% cases). However, we did not recover any *Epidermophyton spp*. Among the *Trichophyton spp., T. mentegrophyte* was the predominant *spp*. (63.5%) followed by *T. rubrum* (35.1%). The male to female ratio of the positive cases was recorded as 63:11. The most effected age group was 21–50 years (64.9%) followed by 1–20 years (28.4%) and above 50 years (6.8%).

## Introduction

Dermatophytosis is a disease condition characterized by the infection of keratinized tissues such as the epidermis, hair and nails. This condition is caused by a group of closely related filamentous fungi commonly known as dermatophytes. *Epidermophyton, Microsporum* and *Trichophyton* are the genera of dermatophytes implicated in superficial mycoses. These organisms are assuming greater significance due to the excessive use of immunosuppressive drugs for controlling serious infectious as well as non infectious conditions. They produce keratinases which degrade the keratin and thus, invade the superficial skin tissue. The infections due to these pathogens are generally cutaneous and restricted to the non-living, cornified layers of the skin. However, in chronic conditions, the fungi may invade deeper tissues, particularly in concurrent infections with other organisms. In general, the dermatophytes lack the ability to invade deeper tissues or organs of the host. The typical infections of dermatophytes are generally referred to as ringworm infections due to their ring like appearance. These infections are also known as ‘tinea infections’ and are named according to the location of the lesions on the body e.g. tinea capitis refers to ring worm infection of the head region. Since these infections are often confused with other skin disorders, it is therefore, necessary to make early laboratory diagnosis for better management of these conditions (Huda et al. 1995). The tinea infections are prevalent globally but they are common in tropics and may reach epidemic proportions in geographical areas with higher humidity, over-population and poor hygienic living conditions (Weitzman and Summerbell 1995; Peerapur et al. 2004). Hot and humid climate of India makes dermatophytosis a very common superficial fungal infection of skin (Niranjan et al. 2012). Various studies have been conducted in different parts of the country including Chennai (Venkatesan et al. 2007), Madhya Pradesh (Pandey and Pandey 2013), Andhra Pradesh (Madhavi et al. 2011; Maruthi et al. 2012), West Bengal (Grover and Roy 2003; Das et al. 2009), Gujarat (Singh and Beena, 2003a; Bhavsar et al. 2012), Chandigarh (Chakrabarti et al. 1992), Karnataka (Reddy et al. 2012) and few other states. The distribution, frequency and the causative agents involved vary from place to place depending upon the climatic, socioeconomic conditions and the population density (Das et al. 2009; Venkatesan et al. 2007).

, the coldest one of Himachal Pradesh by Deshmukh et al. 2010. The study suggests that these organisms are quite tolerant and have the potential to adapt to various biotic and abiotic factors. The prevalence of onycomycoses to the tune of 20% each among farmers and office workers has been reported from Shimla, Himachal Pradesh (Gupta et al. 2007) and the dermatophytes implicated in the study were: *T. rubrum*, *T. mentagtophyte* and *T. verucosum.* Bhagra et al. (2013) reported *Microsporum gypseum* in tinea corporis in a patient with acquired immunodeficiency syndrome at Indira Gandhi Medical College, Shimla. This dermatophyte is uncommon in this tenia condition and represents atypical dermatophytosis. The incidence of dermatophytosis has also been reported among bovines in the Kangra valley of Himachal Pradesh (Chahota et al. 2000). The availability of scanty data on the prevalence and some associated epidemiological factors of dermatophytosis in the state of Himachal Pradesh prompted us to take up the present study which utilizes conventional methods of isolation and identifications of dermatophyte species from superficial mycoses in human patients.

## Methodology

The present study has been conducted on the individuals who visited the skin outpatient department (Skin OPD) at Indira Gandhi Medical College (IGMC) Shimla, Regional Hospital (RH) Solan and Employee’s State Insurance (ESI) Hosptal, Parwanoo, in the state of Himachal Pradesh. While collecting samples, the following inclusion and exclusion criteria were adopted.

The patient visiting the Outpatient department in the hospital settings showing lesions typical of dermatophytosis based on the judgment of the clinician were eligible to participate in this study. Age limit and sex bias were not used and patients of all age groups and both the sexes were included. The exclusion criteria included: use of antifungal therapy (oral as well as topical) within 2–3 months prior to the commencement of the study; presence of serious underlying systemic conditions as adjudged inappropriate by the clinician for inclusion in the study; other infections bacterial as well as fungal in the skin folds and nails such as paronychia etc. were excluded.

The research project SUIEC/12/04 was approved by the Institute Ethics Committee through its letter no. SUBMS/IEC/12/45, dated 19^th^ March, 2012.

### Collection of samples

A total of 202 samples in the form of skin, hair and nails were collected randomly in batches from out patients with different tinea conditions at Shimla, Solan and Parwanoo. These conditions included: Tinea corporis, T. capitis, T. cruris, T. pedis, T. unguium, T. faciei, T. manuum and T. gladiatorum. These conditions were diagnosed by the clinician himself while examining the patients and the same were processed in the laboratory for dermatophytes. For obtaining the samples aseptically, the infected areas or lesions were wiped with 70% ethanol in order to remove the dirt and environmental contaminants. Skin and nail scrapings, hair alongwith follicles were collected from advancing margins of the lesions in sterile plastic containers (Sterile Uricol: Himedia) with the help of sterile scalpel/ tweezers. The information about the applications of antifungal therapy was obtained through inquiry from the patients since the clinician asked them to produce the outpatient chit if any treatment was taken during past 2–3 months. Also, the other information regarding immunosuppressive/immunocompromised state including co-infection with HIV and other conditions such as diabetes was recorded in consultation with the clinician. In addition, the sex and age of infected patients were noted down. The samples were transported to and processed at the Microbiology laboratory of the Shoolini University at Solan.

### Examination of direct KOH mount

Hair follicles, scrapings of skin and nails were treated with 40% KOH for 10 minutes, mounted on a glass slide and examined under microscope for the presence of fungi under low power of magnification. The positive samples were processed for the isolation of the dermatophyte species on Sabouraud’s Dextrose Agar (SDA, Himedia).

### Isolation of dermatophytes

The samples were cultured on the Sabouraud’s Dextrose Agar (SDA, Himedia) containing Cyclohexamide (0.05%) and chloromphenicol (0.004%) under sterile conditions. The plates were incubated at 30°C for four weeks and monitored for the growth. Dermatophytic growth was picked up with L- shaped inoculating needle and streaked on SDA slants. The colonies on the slants were examined for their morphology, texture and pigmentation (obverse and reverse) etc. The confirmation was done by microscopic examination of the stained preparations as described below.

### Identification by microscopy

Colony of each isolate was stained in Lactophenol Cotton Blue (LCB) and observed under low (10× lens) as well as high power (40× lens) of light microscope. The identification was based on features such as organization of hyphae (pencil shaped, spiral, pyriform, septations etc.), microconidia and macroconidia (tear shaped, drop like, spherical, in bunches, abundance or rare etc.). *Trichophyton rubrum* (ATCC-28188), *T. mentagrophyte* (ATCC-18748) and *Microsporum gypseum* (ATCC-24102) obtained from Post Graduate Institute of Medical Education and Research (PGIMER) Chandigarh, were included as standard strains in the study.

### Urease test

This test is used as an adjunct to the microscopic examination for the differentiation of dermatophyte species since most of them have the ability to produce urease enzyme which hydrolyses urea.

## Results

**Figure 1:**
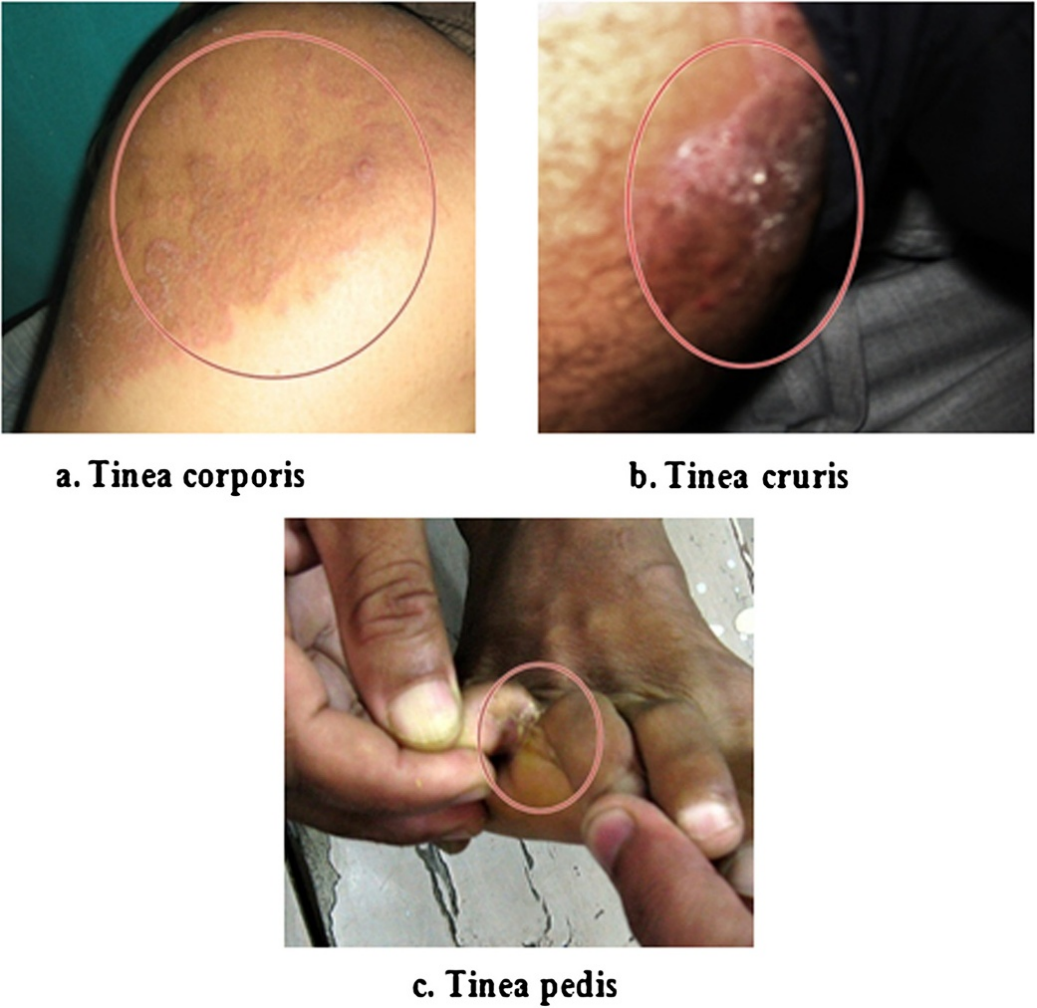
**Ring worm infections in human patients. a**. Tinea corporis in 27 years old male patient at Regional Hospital, Solan (ring shaped, raised, erythematous lesions with distinct margins are visible) **b**. Tinea cruris in 49 years old male patient from ESI Hospital, Parwanoo (typical lesions of tinea are visible on groin region). **c**. Tinea pedis in 46 years old male patient from ESI Hospital.

**Figure 2:**
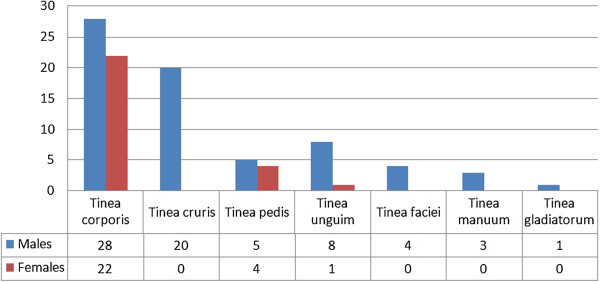
**Details of culture positive samples among male and female patients of dermatophytosis.**

Of the 202 samples analyzed in cultural examination, 74 (36.6%) were found positive for dermatophyte species (Table 1). Among different tinea conditions, Tinea corporis 29/74 (39.1%) figured at the top (Figure 1) followed by Tinea cruris 20/74 (27.0%) and Tinea gladiatorum 1/74 (1.35%) for culture positivity (Table 1; Figure 2).

**Table 1:** **Details of culture positive samples recovered from patients with different ring worm infections**

Tinea infection (Types)	Samples examined (nos.)			KOH positive samples	No. of culture positive samples		
	Total	Males	Females		Total	Males	Females
**Tinea corporis**	**61**	**43**	**18**	**61**	**28**	**22**	**6**
**Tinea crusis**	**35**	**34**	**1**	**35**	**20**	**20**	**-**
**Tinea pedis**	**34**	**21**	**13**	**34**	**9**	**5**	**4**
**Tinea unguium**	**47**	**24**	**23**	**48**	**9**	**8**	**1**
**Tinea faciei**	**7**	**6**	**1**	**7**	**4**	**4**	**-**
**Tinea manuum**	**8**	**5**	**3**	**8**	**3**	**3**	**-**
**Tinea gladiatorum**	**1**	**1**	**-**	**1**	**1**	**1**	**-**
**Tinea capitis**	**8**	**1**	**7**	**7**	**-**	**-**	**-**
**Tinea barbae**	**1**	**1**	**-**	**1**	**-**	**-**	**-**
**Total**	**202**			**202**	**74**	**63 (85.1%)**	**11 (14.9%)**

### Isolation and Identification of Dermatophyte spp

**Figure 3:**
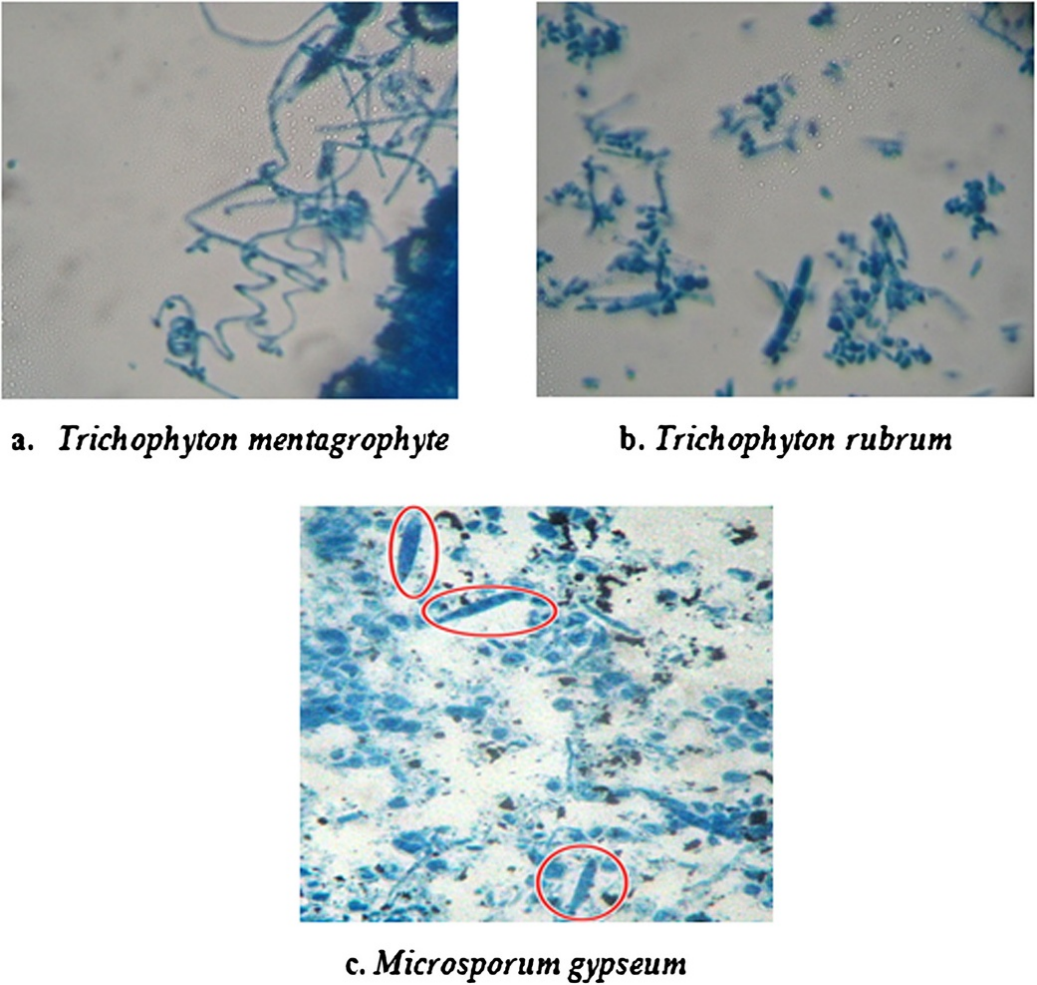
**Microscopic examination of stained preparation of dermatophyte spp.** (Magnification 40 × 10 ×) **a**. Spiral hypae of *Trichophyton mentagrophyte* **b**. Pencil shaped septate hypae of *Trichophyton rubrum* **c**. Pyriform, septate hypae of *Microsporum gypseum* are visible in the figures.

*Trichophyton* species were implicated in 98.6% (73/74) cases while *Microsporum* species was detected only in 1.35% cases. However, none of the *Epidermophyton* species was recovered in the present study. Among the *Trichophyton spp., T. mentegrophyte* was the predominant organism (64.9% cases) followed by *T. rubrum* (35.1% cases) (Table 2). The identification of these dermatophyte species was based on cultural characteristics, growth rate, texture, colony size and pigmentation produced on obverse and reverse sides of SDA slants. *T. mentagrophyte* grew rapidly (3–5 days) on SDA, the growth was powdery to fluffy, cream to white on obverse and yellow to brown on reverse. On microscopic examination, well septate spiral hypahe with numerous spherical microconida were visible (Figure 3a). *T. rubrum* grew relatively slower (10–15 days), the growth was powdery to velvety with reddish tinge on obverse and rusty brown to deep red on the reverse. Well septate, pencil shaped hyphae with numerous spherical microconidia along with macroconida were visible on microscopic examination (Figure 3b). *M. gypseum* grew rapidly (3–5 days), the growth was powdery to granular with rosy pink on obverse and yellow to brownish on reverse. As shown in Figure 3c, pyriform septate hyphae were visible and microconidia were not seen as this organism rarely produces microconidia. *T. mentagrophyte,* the predominant species was found associated mainly with Tinea corporis 40.4% and Tinea cruris 25.5%. However, it was seen in all other tinea conditions also Table 2.

**Table 2:** **Association of Dermatophyte spp. with different ‘Tinea’ conditions**

Dermatophytes	Tinea corporis	Tinea crusis	Tinea pedis	Tinea unguium	Tinea faciei	Tinea manuum	Tinea gladiatorum	Total
***Trichophyton mentagrophyte***	**19**	**12**	**5**	**7**	**2**	**1**	**1**	**47**
***Trichophyton rubrum***	**8**	**8**	**4**	**2**	**2**	**2**	**Nil**	**26**
***Trichophyton gypseum***	**1**	**Nil**	**Nil**	**Nil**	**Nil**	**Nil**	**Nil**	**1**

Among the culture positive cases, 85.1% were of males and 14.9% were of female patients. It was also observed that 64.9% patients fell in the age group of 20–50 years while 28.3% and 6.8% patients were in the age group of 1–20 years and > 50 years respectively (Table 3).

**Table 3:** **Details of sex and age group of patients of dermatophytosis examined**

Male patients			Female patients			No. of patients lie under the age group of					
(M)			(F)								
Total	+ ive	(%)	Total	+ ive	(%)	1-20 years		21-50 years		51 years and above	
						Males	Females	Males	Females	Males	Females
140	63	45.0	62	11	17.7	19	1	39	10	5	0

## Discussion

The present study highlights the clinical pattern and prevalence of different dermatophyte species implicated in different tinea/ringworm infections in parts (Shimla, Solan and Parwanoo) of Himachal Pradesh. The climatic conditions of Himachal are favorable for the development of superficial mycoses (Deshmukh et al. 2010). In general, hot and humid environment of the tropical and sub-tropical regions are best suited for the dermatophytic infections which have been reported from different parts of India. There is a huge variation in the climatic conditions of Himachal Pradesh due to variation in altitude (450–6500 meters). The climate varies from hot and sub-humid tropical (450–900 meters) in the southern low tracts, warm and temperate (900–1800 meters), cool and temperate (1900–2400 meters) and cold glacial and alpine (2400–4800 meters) in the northern and eastern high elevated mountain ranges. Shimla lies in the south-western ranges of the Himalayas. It is located at 31.61°N 77.10°E with an average altitude of 2397.59 meters (7866.10 ft) above mean sea level. Solan city is located at 30.92°N 77.12°E. It has an average elevation of 1502 meters (5249.34 ft). Parwanoo is located adjacent to Kalka, Haryana (30.83°N 76.95°E) which is quite hot during summer and humid during rainy season. Being hilly state, the three cities chosen for the study have relatively higher population density consisting primarily of farmers and a large proportion of the construction workers/laborers particularly in Parwanoo which is an industrial area. Besides the climatic conditions favorable for the growth of dermatophytes, other factors such as the migration of laborers, workers and tourists frequently visiting this region, overcrowding, unhygienic life style of the community with low socio-economic background might contribute to the development of dermatophytosis in this region of the state. Of the total 202 cases of superficial skin infections examined, only 74 (36.63%) were found positive in culture. Of the positive cases, 85.1% were males and rest females (Table 1). Such higher prevalence in males has been reported in India as well as other countries of the world by several researchers (Singh and Beena 2003a; Balakumar et al. 2012). This may be due to the differences in occupational exposure of both the sexes as males are more involved in construction and other works. Further, although patients of all ages were susceptible to dermatophytosis but most (64.9%) belonged to the age group of 21–50 years. Almost similar observations have been made by others. They have reported highest prevalence in the age group of 21–30 years (Sarma and Borthakur 2007; Patel et al. 2010). The probable reason for higher prevalence in this group could be that the individuals in this group are often most active because of their involvement in the outdoor activities such as studies, jobs etc.

Various tinea conditions in the present study were diagnosed by the clinician himself based on the clinical presentation. Tinea corporis was the most common clinical condition observed in which various exposed parts of the body are affected followed by tinea cruris in which groin and surrounding areas are affected. The clinical picture of these conditions is presented through Figure 1. Similar observations have been made by other researchers (Venkatesan et al. 2007). Tinea conditions are consequence of exhaustive physical work and prolonged exposure to sun leading to excessive sweating. In addition, the tight fittings and synthetic clothing particularly in males provide damp, sweaty and warm skin conditions. All these factors favour the growth of dermatophytes (Ranganathan et al. 1995; Singh and Beena 2003b). Tinea pedis and tinea unguium might result from wearing of socks and shoes for a long period providing damp conditions especially in inter-digital spaces.

In the present study, *Trichophyton mentagrophyte* was the predominant dermatophyte (63.5%) involved followed by *T. rubrum* (34.6%). *Microsporum gypseum* was involved only in 1.35% cases. We, however, did not observe any involvement of *Epidermophyton spp.* in the present study*.* Interestingly, we found *T. mentagrophyte* as predominant species followed by *T. rubrum*. This finding is contrary to the observations of others in which a reverse trend has been reported (Balakumar et al. 2012; Patel et al. 2010; Pandey and Pandey 2013). The plausible explanation for this can be seen in the fact that *T. rubrum* is generally linked to chronic dermatophytosis (Aya et al. 2004). However, we do not have exact data about the chronic cases of dermatophtosis as they were excluded on the basis of history extracted from the patients. Therefore, the low proportion of *T. rubrum* might be involved in acute superficial mycosis. Besides, the use of effective and prolonged antifungal therapy to treat the patients might have reduced the incidence of *T. rubrum* in this region. Further, this organism is a slow growing organism, there is a possibility that other dermatophyte species might overgrow or mask the growth of *T. rubrum* while attempting isolation. PCR amplification directly from the samples could be a better tool to prove this. We are developing molecular diagnostic tools for rapid and early identification of these pathogens which in combination with conventional methods would facilitate early management of dermatophytosis. Several researchers have reported the association of non-dermatophytic and other fungi with dermatophytosis world over (Havlickova et al. 2008; Enemuor and Amedu 2009; Prasad et al. 2013). Such studies are now under way in our laboratory.

It may be concluded from the present study that the climatic conditions of Himachal Pradesh favour dermatophytosis in the population, Tinea corporis was the most frequently encountered clinical condition followed by tinea cruris. *T. mentagrophyte* was implicated as the predominating species followed by *T. rubrum* and *M. gypseum.* Unhygienic conditions among low socio-economic group, frequent migration of laborers, workers, frequent visits of tourists to this region may be some of the contributing epidemiological factors. Although, the present study is a random study that focuses primarily on the prevalence of different dermatophyte species in the state of Himachal Pradesh, more systematic study covering larger population and over a longer period of time would give a better insight into the epidemiology of dermatophytosis in the state.

## Consent

Written informed consent was obtained from the patient for the publication of this report and any accompanying images.
